# Governmentality Versus Community: The Impact of the COVID Lockdowns

**DOI:** 10.1007/s42413-023-00189-7

**Published:** 2023-05-09

**Authors:** Claire Wallace, Lucia Mytna-Kurekova, Margarita Leon, Jacqueline O’Reilly, Constantin Blome, Margarita Bussi, Becky Faith, Mark Finney, Janine Leschke, Chiara Ruffa, Emma Russell, Mi AhSchøyen, Matthias Thurer, Marge Unt, Rachel Verdin

**Affiliations:** 1 University of Aberdeen, Aberdeen, Scotland; 2 Slovak Academy of Sciences, Bratislava, Slovakia; 3 Universitat Autonoma Barcelona, Bellaterra, Spain; 4 University of Sussex Business School, Falmer, England; 5 University of Sussex Business School and Louvain School of Management, Falmer, England; 6 University of Louvain, Louvain, Belgium; 7 University of Sussex Business School, University of Louvain, Louvain, Belgium; 8 Emory and Henley College, Emory, USA; 9 Copenhagen Business School, Frederiksberg, Denmark; 10 Uppsala University, Uppsala, Sweden; 11 University of Sussex, Falmer, England; 12 Norwegian Social Research, Oslo Metropolitan University, Oslo, Norway; 13 Jinan University, Guangzhou, China; 14 Tallinn University, Tallinn, Estonia; 15 University of Sussex Business School, Falmer, England

**Keywords:** Community participation, Community well-being, Governance, Technology and well-being

## Abstract

The COVID lockdowns were characterised by new forms of governmentality as lives were disrupted and controlled through the vertical transmission of biopolitics by the state. The paper considers how this was experienced by academics in 11 different countries through analysis of diaries written during the first lockdown. The paper asks if communities can offer an alternative to governmentality by looking at three levels: the national, the neighbourhood and the personal. Whilst at a national level the idea of community was instrumentalised to encourage compliance to extraordinary measures, at the local level community compassion through helping neighbours encouraged horizontal connections that could offer a “space” within the dominant logic of governmentality. At the level of personal communities, the digitalisation of social relationships helped to create supportive networks over widely dispersed areas but these were narrowly rather than widely focused, avoiding critical discussion.

## Introduction

The Coronavirus lockdowns forced people to adapt their community life as travel was restricted and public and leisure services were withdrawn. In Foucault’s (2003) framework, this meant new forms of territorial governmentality and the reshaping of the relationship between individuals and the state as the biopolitics of emergency were imposed. The lack of the usual places of interaction such as cafes, bars, pubs, places of worship and community halls meant that horizontal communication with friends, family and neighbours was restricted as the normal fabric of daily life was torn apart(Colombo, 2020). At the same time, vertical forms of communication from above were reinforced through rapidly implemented and perpetually changing instructions and regulations from the state (Bigo, 2021; Pellizzoni & Sena, 2021). Digital communications further reinforced the pervasive power of biopolitics through ubiquitous bio-surveillance(Agamben, 2021). Indeed, digital communications became one of the only ways that the horizontal connections of community could be carried on, so that they took on enhanced importance. This paper, drawing upon auto-ethnography by high digital density households from 11 countries, considers how community was defined and redefined through the pandemic.

Foucault’s concept of govenmentality is highly relevant to how the pandemic played out for actors at a micro-level of social life (Foucault, 2003). This reinforced the national field of power at one level as countries enacted extraordinary powers within territorialised national boundaries, including night time curfews, requiring permission for leaving home, compulsory mask wearing and restricting social contacts (Bigo et al., 2021). At the transnational level, the field of power included international institutions such as the World Health Organisation or the European Commission, which reinforced the technocracy of control and chivvied deviant leaders such as Trump (USA), Orban (Hungary) and Bolsonaro (Brazil) into line. As the pandemic progressed, “liquid surveillance” enabled by mobile digital technology to manage vaccinations, widespread testing, contact tracing and compulsory quarantines expanded. Voluntary cooperation from reflexive individuals through their “subjectification” and “the active engagement of individuals in their own surveillance” (Bigo et al., 2021, p.19) became widespread as an extension of governmentality(Sylvia, 2020). It is not clear where the space for resistance might have appeared in this ubiquitous and repressive paradigm.

During COVID lockdowns, " biopolitics " was a technical logic by which government by experts replaced dominated and even overrode even the state’s economic interests. This led to the daily litany of death and infection tallies, illustrative of what Agamben (2021) has called the “thanatopolitics” of the management of the population that distinguished the “making live” from the “letting die” (Foucault, 2003). Politics was reduced to “bare life” (Agamben, 1998). For example, some more vulnerable groups such as residents of care home or elderly isolated people were sacrificed to keep others alive and front-line workers such as nurses, care workers and cleaners were allowed to continue working, even where appropriate safety precautions were not fully available.

The problem of how this relates to communities is problematic. Communities filter and reproduce these biopolitics as part of the micro-level of social relations within a society. But communities can also represent an alternative to biopolitics by opening horizontal channels of communication. Community as a “coexisting multiplicity” is important as a source of belonging as people search for meaningful attachments to anchor their identities (Delanty, 2003; Mulligan, 2015). Community is therefore seen as a potential source of social cohesion and meaning. Community became even more important under conditions of COVID lockdown as people who were forcibly isolated and cut off from regular communications, reached out for connection. At the micro-level, a community can cover a variety of shared and meaningful horizontal social relationships, including ones connected to a given place (Kuecker et al., 2010). But do communities offer an alternative to governmentality?

A number of studies documented the turn to community during the COVID-19 pandemic through the mobilisation of community volunteering at a local level (Carlsen et al., 2021). Volunteers in Denmark provided both economic and social support of various kinds at a local level, taking over where the state and private sector had retreated, although this “imposed volunteering” had uneven gender dimensions (Andersen et al., 2022). One study described this as a form of “bottom-up social innovation” (Grasso, Klicperova-Baker et al., 2021). Data from the comparative surveys carried out by the European Foundation for the Improvement of Living and Working Conditions indicated a relatively large number of people volunteering in many countries during the pandemic (Eurofound, 2021). In the UK, the Carnegie Trust documented various examples of local initiatives mobilising under lockdowns (Coutts, 2021). However, others suggested that this was not universal and that more deprived communities were less able to develop this kind of mobilisation (Borkowska & Laurence, 2021). The extent of community organisation depended upon local leaders being in place with the capabilities to manage a bottom-up approach. Hence it was often done through local religious organisations with a ready-made set of community leaders, although this might have had uneven consequences.

In the governmentality paradigm, local communities are seen in terms of “responsibilisation” as they took over aspects of support for citizens but using the same neoliberal paradigm of self-help, targeting, under conditions of austerity. Hence, the idea of the neo-liberal individual was extended to communities. For example, Lynda Herbert-Cheshire analyses how the tradition of community autonomy in rural Australia becomes “governmentality at a distance” (Herbert-Cheshire, 2000). Fraser (2020) shows how community development workers adopted the language of the new managerialism. However, horizontal forms of communication could also challenge vertical (top down) lines communication as Rolfe (2018) and Fraser (2020) both acknowledge. Indeed, Rolfe suggests that community empowerment can take “positive sum” directions through working together with the state rather than the “zero sum” measures with state responsibilities simply relegated to local communities, some of which may lack the capacity to respond. Is this what also happened during the COVID lockdowns?

The idea of community has been further transformed by digital communications offering new possibilities for social connection and identity which can stretch across the globe. Wellman has argued that the digital communities might differ from others because they form looser and more dispersed networks (Rainie & Wellman, 2012). Arguably they are also more transient in nature because they can both materialise and dematerialise remotely at the click of a mouse. And yet this has also facilitated place-based relationships within a locality (Hampton & Wellman, 2003; Wallace & Vincent, 2017; Wallace et al., 2017). Digital groups create new kinds of neighbourhood conviviality. The forced digital turn induced by lockdown helped to reinforce and reconstruct virtual communities as digital communication became the only way to communicate.

In this paper, we explore how community has been transformed under conditions of coronavirus lockdown at three different levels: national, local and personal. We argue on the one hand for the increased salience of community, both physically and virtually on account of digital communications becoming one of the main ways of connecting people, but on the other hand we observed the increased territorialisation of community, which took different forms at different levels.

## Context

Biopolitics took different forms in different countries, but with many similarities as a consensus emerged about how the virus was to be managed as technical rationality. Most countries instituted lockdowns of various kinds during the first outbreak of Coronavirus in Spring 2020. However, the nature and extent of the measures varied. Sweden stands out as a country where strong lockdown rules were not applied and compliance was largely voluntary rather than mandatory. For example, whilst all the countries in our sample (apart from the US) instituted a “stay at home” policy, in Sweden people were expected to assess the risks themselves. There was full closure of places of worship, non-essential shops and businesses, schools and day care centres, pubs, restaurants, bars and cafes, gyms and sports centres in every country except for Sweden. Hotels and other accommodations were closed in most places. All events were banned, there were restrictions on outdoor and indoor gatherings (although the number allowed to gather varied) and private gatherings. In some countries (Belgium, Denmark and the UK) there were even restrictions on the social circle within which people were allowed to interact, creating “social bubbles” for a limited number of people. Teleworking was encouraged everywhere and many countries made workplace adaptations for those places that remained open. There were universal quarantine restrictions for all international travellers (making travel inconvenient and expensive) and most countries forbade people from going abroad unless there was a good reason. In most countries, public transport was stopped or severely cut back. The term “social distancing” was introduced, with people told to remain two metres apart (later reduced to one metre). Mask-wearing in public places was made mandatory in all countries apart from Sweden (European Centre for Disease Prevention and Control, https://www.ecdc.europa.eu/en). In the US, compliance was variable state by state.

Here we focus mainly on the first lockdown, which produced the most significant dislocation to social life, but subsequent lockdowns varied in duration and the extent. While the specific lockdown conditions differed across the countries covered in this paper in their duration and stringency (Hale et al., 2020), they all entailed significant and often abrupt disconnections from the old forms of interaction, work organisation, family routine and social engagement. Political debate was replaced by technocratic control as health experts stood alongside politicians during public announcements. In China, there was one of the most extreme reactions with “zero tolerance” policies to stamp out the virus in the places where it was discovered. This led to severe economic consequences for China’s economy and considerable hardship at an individual level as infected people – or people who had been in contact with infected people – were shipped out to special centres and detained indefinitely (Ling, 2022).

The pandemic resulted in a kind of “COVID nationalism” (de Kloet et al., 2000) as countries strived to keep resources for their own citizens, borders were re-erected in the Schengen zone and reinforced elsewhere and trans-border networks severed (Opilowska, 2021). To reduce national differences and conflicts, the EU set out to collectively coordinate the purchase of medical materials, develop a vaccination programme and provide a redistributive financial rescue package. However, the result was slow and cumbersome with the EU being criticised by individual countries and international organisations. At first the test and trace programmes were generally national ones rather than internationally coordinated - although later replaced by EU level certification. Nevertheless, internecine competition between countries repeatedly erupted, especially once vaccines were introduced.

In general, there was a decline of trust, increased anxiety and attenuation of social networks, with steep drops in mental health reported across Europe, particularly affecting young people (Eurofound, 2021), and a sense of risk pervaded all social interactions (Zinn, 2021). Face-to-face social contact was seen as hazardous and frightening - threatening established forms of community participation. However, we do not know how people in practice experienced this as part of their daily lives.

The key questions are, therefore: how did people at different community levels experience the COVID lockdowns? How did the turn to the digitalisation of communications affect these community ties? Was community mobilisation an alternative to governmentality or simply an extension of it?

## Methods for Research

It was difficult for researchers to conduct original research during the COVID lockdown conditions. Face-to-face interviews were not allowed in many countries. Therefore, less conventional methods of data collection were called for and here we developed a forum for “auto-ethnography” (Wambura Ngunjiri et al.,^2010^, Chang, 2008). It involved writing and self-reflexive analysis of one’s own diaries and that of others. The authors were asked to reflect upon several topics, namely work, children, older family members, self-care and mental health and government and employer behaviour, and to consider the aspect of digital communications in their reflections. The sense of community emerged as a cross-cutting issue across the diaries. All participants are included here as co-authors.

The diaries were coded and analysed using a combination of NVivo and Framework (Spencer & Ritchie, 1994) to identify common themes. This enabled a form of research whereby themes could be continually created or merged until a common set of parameters could be identified and the data set shared with other NVivo users. The analysis formed part of an ongoing discussion with participants in the project. The exercise was organized in an organic and decentralized manner. Each diarist decided what to include and was able to read and comment upon other entries uploaded into a Dropbox file.

The research presented here originated in a network of 14 social scientists from 11 countries organised in March 2020 through the Digital Future of Work Research Centre. Social Scientists from different parts of the world were invited to join the network. Countries included Belgium, China, Denmark, Estonia, Germany, Norway, Slovakia, Spain, Sweden, UK and USA. Each participant kept a diary during the first lockdown from March until June 2020 when the first lockdown was imposed. There were several virtual meetings of the group and the various subgroups during this time and afterwards. The households they belonged to were characterised by high levels of digital density, owning many devices and being familiar with online communication already before the pandemic. Diaries were updated in Spring 2021 with winter reflections from the intervening period.

Country codes replaced all participant names to protect their identities – China CN, Spain ES, Slovakia SK, Norway NO, Estonia EE, Denmark DK, Sweden SE (two from Sweden SE1 and SE2), USA US, Germany DE and Belgium BE. In the UK there were three participants, one from Scotland (SC) and two from England (EN1 and EN2). These pseudonyms do not refer to the nationality of the participants since people were often located in countries other than their own. This may have helped their observational powers but may also have increased their sense of isolation. Most of the participants were females but those from China, US and Germany were males.

### Territorialisation of Governance at a National Level

Diaries showed that the territorialisation of vertical power at a national level was managed by building upon a sense of emergency with a need for exceptional responses. Frequently likened to a response to war or natural disaster, obedience was induced through a pervasive sense of fear and anxiety (Agamben, 2021). The diaries described how governments imposed stringent measures on populations’ daily behaviour and freedoms, which left them reeling. ES mentions that even the woods in her Spanish homeland were cordoned off with police tape to stop people from walking there. Many of the countries in which our diarists lived imposed overnight curfews. In Britain and the US the more liberal regimes under populist leaders (Trump and Johnson) at first rejected the strong lockdowns imposed on most European countries, but before long, restricting personal movement was seen as the only way to combat the disease. It seems that everywhere except for the same technocratic ideas for control were adopted – although to a lesser extent in Sweden. In the UK, the restriction of social life to “bubbles” relied more on self-policing through voluntary subjectivisation but in other countries such as China or Spain, measures were enforced by real police on the streets.

Our diarists reflected on the development of a “COVID nationalism” as each country developed variations on the response. They reported obsessively tuning into daily news broadcasts and learning of the lates regulations and death tolls as information horizons shrank. Diarists fervidly followed public broadcasts as the top-down vertical spread of power replaced the daily conversations and encounters that existed previously. Thus, there was a radical re-focus of the idea of national community with daily top-down instructions controlling every day behaviour in the “thanatopolitics” of death and infection.

In general, people complied with this severe curtailment of their freedoms, resulting in economic hardship for many people. How was this managed? Here the idea of community spirit could be mobilised through a call to national unity and community altruism to sustain this national effort (Brooks et al., 2020). This resulted for the most part in panicked adaptation rather than resistance as our diarists were discouraged or forbidden from going to their offices and had to move their teaching and research online, often at considerable expenditure of time and effort. “We are all in this together” was a general refrain, overlaying significant regional, social and demographic differences in the prevalence and impact of the disease. Emblematic of this was the 8pm applause from doorways and balconies in appreciation of the health workers, which the diarists welcomed. Beginning in Italy, it spread to Spain where clapping became a daily ritual that brought people together in an act of collective solidarity. Clapping from doorways also became a Thursday night ritual in the UK as people applauded the otherwise underfunded and overworked healthcare staff who were risking their lives daily. Our diarists shared news of people playing instruments, singing or dancing in front of their houses and screens, spreading these activities through social media.

Social solidarity and community social cohesion was demonstrated in other ways too. Diarists described how In Britain, children painted rainbows and mounted them in windows as a gesture of support for the National Health Service. People also painted stones and messages of support on pieces of wood from Easter time 2020 onwards. All three UK diarists mentioned the pleasure of looking for rainbows and support messages in windows and alongside walking paths. Altogether calls to the “national community” involved the deployment of national myths, symbols and heroes recalling other times of national emergency. This appeal to altruism and self-sacrifice diverted attention from the inadequacies of government responses. This “national spirit” built upon the populist nationalising tendencies that had already been emerging in European countries and elsewhere (James & Valluvan, 2020).

Sweden adopted the “herd immunity” approach on the advice of the Chief Medical Officer and relied on good citizen self-control to ensure social distancing and adherence to safe behaviour. Yet the Italian diarist in Sweden (SE1) was very critical of government policies.

*Sweden smug and nationalistic….. People are proud of their public health agency and even when now things are starting to shake a little the support to the Swedish public health agency is still rocket high…. Me and several other friends have been very sad and disappointed. The very idea that people on average are acritical, that even people in academia have been scared (like me and many others) to speak up makes me question the very nature of this society. The Swedish strategy to Covid has been to me an…unprofessional careless expert rule ….combined with a narcoleptic sleepy public and mediocre journalists unable up until very recently to challenge conventional wisdom.*

As horizontal communication links were severed or severely constricted, it emerged only slowly that the biopolitics of death among ethnic minorities and other disadvantaged populations were disproportionately high (Bertocchi & Dimico, 2020). The pre-eminence of national news sources and lack of opportunity to travel might have narrowed focus on the national experience. However, this call to an idealised national community, helped people accept the radical change in their lives and economic hardship (for some). It glossed over the widening inequalities between those who could work at home benefitting from living in high digital density households and those who lost their jobs and started to suffer real hardship or were more digitally excluded. Some of these tensions are evident in the diary from Slovakia.

*Different pressures are emerging – the voices of the large and strong are heard. Some say we should support banks, others point to large businesses, but I hear very little of caring about those who have fallen through the net – and are not covered by social assistance as we pushed so many people out in the past years. They managed (previously), because labour market was strong, but from day-to-day they lost their opportunities for temporary, black-market, migration jobs. One Roma settlement is in quarantine - they are short of food, and cannot leave the settlement… How is the government going to reach them?*

Hence, at a national level a territorialised community was reinforced through the erection of national borders and the focus on national resources. Furthermore, the call to solidarity and social cohesion meant that the idea of national community was continually reinforced. This helped to silence opposition and restrict channels of communication that might have led to alternative voices being heard – at least during the first lockdown.

### Territorialisation of Governance at the Local Level

The local community took on a special significance because of travel restrictions: it was where people now spent all their time. As a result of lockdowns, public transport was curtailed, car driving discouraged, and many people in need of help with shopping, getting prescriptions from the doctors and so on, especially if they were elderly or self-isolating, had to depend on local volunteers or neighbours.

This local community also took a digital form. There was an extraordinary community response, often spontaneous and bottom-up, as Facebook pages or WhatsApp groups based on neighbourhoods were set up. Neighbours called on elderly or shielding people and set up telephone networks. This kind of response was found in many of the diaries. In North-East Scotland, *The Grampian Hub*, coordinated police, local authority, emergency and other services including NGOs such as the Red Cross and volunteers. It involved 350 groups and 4000 individuals. Police officers were redeployed to staff telephone lines and put people in touch with relevant local services. Around 9000 people were supported with food boxes, shopping, prescription collection and so on (from a population of around 500 000). Indeed, there were many more volunteers than were needed by a ratio of about three volunteers offering themselves for each one needed in this area (from interviews with community leaders). However, rather than being just another example of “governmentality at a distance” it represented a spontaneous eruption of altruism and generosity. We could see it as opening a “space” for horizontal communications within the closing walls of the governmentality prison.

In a village in Scotland, where SC lived, a “community hub” was organised through social media and coordinated by the resident Church of Scotland minister. This involved organising volunteers on a street-by-street basis delivering services such as prescription collection, shopping and dog walking. Twice as many people volunteered to help as needed, so most were never called upon. The community hub also organised a local food bank for those who needed it in the Church Hall and this included clothes, toilet paper, children’s games, plants and other things that might be in short supply donated by members of the local community. The local Facebook page expanded its membership and its remit from just being for “parents in the village” to including a much wider community and many more people now called the “Village Community Group”. Local social media sites became the way in which information was spread rapidly across the community. Local businesses were able to advertise their lockdown services in this way. With a population of 2700, the village community FB page attracted 2893 members - so the social media site was even bigger than the community’s population.

Whilst this formal style of horizontal organisation was built upon existing church and food bank structures, in other countries this generosity took a more informal turn. The Spanish diarist noticed:

*We have seen lots of very small initiatives (at neighbourhood level) to help the elderly with their shopping, holding conversations over the phone, etc. I think you get a sense of how fragile ageing is.*

ES also mentioned the informal support that blossomed in the residential blocks as healthier and younger people cared for older ones. SK noted that whilst in her residential neighbourhood, there had been interaction among neighbouring children, this disappeared with lockdown and local relationships dwindled to the next-door neighbour. Nevertheless, SK baked and delivered cakes to a neighbour during the period of stringent lockdown, by leaving them outside the door. In both Spain and Slovakia small acts of kindness were mentioned in relation to children’s birthdays. In Spain, ES describes collective (socially distanced) singing in the neighbourhood as people gathered on their doorstops to express birthday wishes.

*It’s very difficult to go through grim days sometimes but the 8pm applause always cheers me up, such an amazing singing of unexpected and improvised “neighbourliness”. This evening we all sang happy birthday to a young girl from the block in front.*

Our participant in China mentioned the activities of local restaurants delivering take away services, as happened also in many European cities. In the Scottish village, a local butcher provided a ready-made meals service for those who could not get out, which volunteers delivered. In the UK, Muslim and Hindu communities rallied similarly around their members.

Even under normal circumstances, neighbourhoods are traditionally more important for certain categories of people such as children and older people, who might be more locally restricted. Many of the children use the same playgrounds and attend the same kindergartens and primary schools locally. Parents in a locality might form clothes and toys exchanges, reinforcing these connections. This certainly seemed to be the case in the suburbs of Bratislava, where SK tells us:

*On Sunday I really had an urge to demonstrate our existence. I suggested to Jan that he draws a picture for Misha, his best friend neighbour. He drew a car…… put it in their mailbox. A great reason to leave the house! Misha loved it. So Jan drew another four pictures (cars and dinosaurs). In the meantime I baked a cake, so I packed some of it, and we went to give it to them. I also promised to another neighbour that I will share with her my old pregnancy clothes…... We just left it in front of the door, with some cake and strawberries. We exchanged messages via WhatsApp – she loves the clothes! I felt good.*

Perhaps because of the localism of children’s play networks, mothers in the neighbourhood tended to organise into support and exchange groups under COVID conditions too. Some participants mentioned WhatsApp groups being created amongst local mothers to enable this. This reassertion of horizontal ties at a local level helped to create a “space” for social action.

Diarists described how more people spent more time in the local community and the streets and walking paths were full of people walking, cycling or socialising (at a distance). The absence of traffic helped this, and social media shared information about new walks and features of the surrounding areas.

*Passed a group of people chatting (at a social distance) in the middle of the street. Normally it would not be possible due to all the cars* (SC)

Altogether then, local neighbourhoods were reinvented as arenas of social life. This might always have been the case for children, this was also increasingly the case for adults who could no longer go anywhere else. Furthermore, the neighbourhood became a focus of self-help organisation during the lockdown facilitated through social media.

Even in China, the most repressive and controlling regime there was room to develop horizontal connections. Here, the courtyard acted as the focus of micro-social life. Our diarist (who was German) described the courtyards in the communal apartment blocks as becoming important meeting places:

*The courtyard is the place to meet other kids (some from the same kindergarten) and parents, the latter is also a relief for us as our social contacts are rather reduced now with some of our friends having old parents to take care of and thus keeping to themselves; others generally being more cautious than we are. The atmosphere in the courtyard is quite special and communal – more talk than usual.*

Many people mentioned the importance of going for walks locally, interacting with neighbours outsides and enjoying the natural environment, including parks, woods, hills and cemeteries. Life slowed down and became more leisurely:

*The amount of sharing on our road of the most random of things has been lovely. It also seems that when people are out they are not as absorbed by their phones as they were previously. People are looking up and enjoying being outside as opposed to just scrolling as they walk. I suppose we are all enjoying being outside and immersing ourselves in the experience in a way that didn’t happen before (EN2).*

*Lots of virtual support. Got to know lots more of my neighbours as a result. Very nice community feel to it and made some good friends. People sharing things on the community group. People seem more present when they’re outside during lockdown. Shift from no contact with strangers to lots more general friendly chat. (SE2)*

However, under conditions of “liquid surveillance” communities were also informally policed by their members. The lockdown can only work if widespread compliance with changing regulation regimes exists. Being at home enabled different kinds of surveillance to emerge by neighbours and others. For example, ES from Spain showed how this can be exacerbated by spreading pictures of “deviants” on social media, which can also be a source of malice and distress:

*Twitter was filled with messages of hate because some pictures were circulated showing families not respecting the safety distancing. The reality can be so distorted on social networks. Whenever we start going out again is going to be more difficult to police in practice so unless we trust each other in our capacity to stick to the rules or I don’t see how we are going to find our way out of this.*

In the UK neighbours took photos of people exercising more than the regulation twice daily and circulated them to shame the transgressors. In rural communities, signs went up to deter tourists or people visiting their holiday homes. The anger was stoked by anxiety about spreading the disease and some uncertainty about interpreting the guidance in this and other instances.

Therefore, it seems that local communities were galvanised as community hubs to help people in the area. They became “communities for themselves” in the words of Jeffrey Alexander (2006). In some countries this took the form of well organised arrangements, like the Grampian Hub, and in others more informal neighbouring. Lockdowns first imposed the focus on the hyper-local, but it enforced people’s reconnection with their dwelling places at the micro-level of community. The online networking groups helped create community as a virtual space within a particular locality. Furthermore, the social control of behaviour also moved to a local level.

### The role of Personal Communities

The third level of community includes connections of conviviality and affinity at personal level (Pahl & Spencer, 2004). These connections were much maimed and distorted by the lockdowns as micro-level communications were minimised or became impossible. However, in high digital density households, people can maintain various links with family, friends, and colleagues of varying intensity. Academic workers are accustomed to communicating through email, video links and skype. They soon adapted the emerging digital platforms such as Zoom or Teams to their personal communities with the possibility of sending pictures and video calls through WhatsApp, providing a new layer of communication beyond just telephoning. They could involve both real-time and non-real time conversations via email or texts. These forms of communication have multiplied exponentially with lockdowns.

Parents observed that young people and children developed their own social media channels (Instagram, Snapchat and TikTok) partly to exclude adults and create their own social worlds. However, digital communications also became important for staying in touch with older relatives and to some extent, the entry into the virtual world was facilitated by inter-generational transmission. This was clearly the case with family relationships during lockdowns and as people could no longer visit relatives further afield, so digital communications became more critical for maintaining these connections. ES tells us that in Spain:

*Grandparents are 600 km away. We talk to them daily, send them pictures,…grandpa is 80 + with fragile health which makes him a high-risk group so we are worried. …..My mom is fine now…, but we talk a lot more often over the phone. I send her pictures, she records poems for the girls, gives them cooking recipes which we then show to her. We promised to visit the moment we are allowed to travel.*

Some diarists described how older family members might have had to be coached through the new digital media, but once they became adept at it, they stayed in touch this way so that in fact the initial fears of losing touch with elderly relatives was replaced by virtual interactions. As EN2 says:

*Have got some of my resistant family members to get their head around Zoom. This involved lots of step by step phone calls but we managed it. Not altogether sure they enjoyed it though?! We’ve managed a couple of calls which has been good. Am really missing family. We’re all going away this Christmas together and finding myself already looking forward to it……I phone them a lot, we Facetime. We are planning on having digital drinks..*

The result of this concern about relatives linked with the possibilities of digital communications means that in many cases, contact was more frequent and intense than previously.

The diarist from Norway, along with many others, described how they set up WhatsApp groups for children and a number of diarists mentioned “Houseparty” as a platform for meeting relatives and friends. The US diarist describes a concert by his children which enabled relatives from across the USA to connect with them.

The online community can help to maintain to a widely dispersed set of contacts. However, casual acquaintances are more likely to be excluded in this process, making new friends is more difficult. For mobile academic families, these kinds of connections can have particular importance. ES was impressed how people sent videos and messages of greeting for her daughter’s birthday from all over the world:

*Today is Maria’s 10th birthday. She won’t have the usual ‘birthday at the park’ with her friends but we will organize her one at home. A week ago, I asked friends and family to send a short video with their wishes, yesterday I spent almost the whole day putting these videos together. Is just amazing, everyone under lockdown in their homes and coming with very creative ways to wish Maria happy birthday: songs, poems, drawings, dancing, magic…so special. In a single video we have travelled around the world. From her close friends who live just a few blocks away from family in other parts of the country but also friends from other places in Europe and America, North and South…she was really amazed. I don’t think these would have been possible in normal circumstances. With our day to day lives it is very difficult to find the time to be creative in this way.*

Therefore, personal communities became de-spatialised and reduced to digital communications. Contacts with the few face-to-face friends and acquaintances that were still allowed were particularly treasured. People developed new styles of conviviality by meeting outside and found new rituals of sociability within the constraints imposed by social distancing. As the Estonian respondent expresses it:

*I go with morning walking with Lena We don’t hug for hello. Weird. Still so great to see another person in personϑ*

Distanced and online relationships took on a new meaning too. Many people stayed in touch with friends in this was important for this highly mobile group of people. Others had social meetings involving drinking or eating together online. Sometimes, this was because there was some anxiety about a network member that was silent or sick. Concerns about mortality did tend focus the mind on what is most important in life. These contacts became very important for maintaining a social life of sorts.

The awkwardness of Zoom or Teams social meetings was mitigated through the use of quizzes, which many people adopted to avoid the one-at-a-time communication by talking heads. Language conversation, music, and exercise classes started to be organised this way. Communication platforms such as Zoom were revealed to be both intensively private but also invasively public, sometimes with hilarious results, when someone standing up at a meeting was wearing only their underpants or a cat walks in front of the screen.

Online communication facilitated the circulation of jokes, memes and photos, and YouTube videos took on particular importance, later joined by TikTok. Shared humour was a way of managing the isolation and mental stress of lockdown. As our respondent in Estonia expresses it:

*….in a way friends and kids sending and showing great Youtube, Instagram and Twitter jokes makes me feel better and connected to the world. People are so creative!*

However, online communications also bring their particular stresses and anxieties. As the US diarist described it, he became quite obsessed with looking at social media every day and:

*So, how are my friends doing this? Or is it that I’m suffering from Social Comparison disorder, that phenomenon wherein heavy social media users’ sense of self decreases and FOMO [Fear of Missing Out] increases, as a result of heavy social media use? Maybe I’m just focusing on those high points in my friends’ lives and that makes me feel especially low? I don’t know.*

Personal communities with scattered friends and relatives could be easily connected using communications technologies such as WhatsApp or Facetime and work-based platforms such as Zoom and Teams. In this respect the lockdowns even fostered multi-layered forms of communication across distances and within localities. Networks became in this way both dispersed and more focused on family and close friends rather than a range of acquaintances or work colleagues. This meant that the space for critical interactions discussion was reduced, which might partly explain the lack of opposition to the new biopolitical governmentality. Whilst “liquid surveillance” might have been widespread, people confined to their homes could also create networks of sociability through digital channels, which helped to open up a “space” for horizontal communications.

## Discussion and Conclusions

This paper has discussed how the new biopolitical governmentalities introduced under the COVID-19 regulations have affected communities at different levels. At the national level, community spirit was instrumentalised to confront the emergency by reinforcing national boundaries, flows of information and resources. There was a highly technocratic response in most countries. This celebration of the national community avoided the confrontation of other problems and silenced opposition as the inequalities generated by top-down imposed social isolation took a while to emerge. The growing body of evidence of widening inequalities both within and between communities (Cotofan et al., 2021; Bonacini et al., 2020) undermined the discourse of a national community where “we are all in it together”. The misery of dying alone in a care home, the explosive stress of locking up families for children and women and the compounded educational inequalities of keeping children out of school only emerged gradually once lockdowns ended.

At a micro level, locally spatialised communities were reinvented through necessity and often reinforced through self-help initiatives. Far from undermining spatialised relationships, digital media - especially social media - tended to reinforce them as a response to emergency. Locality and place have become more important as a source social support. Online communications within localities have been used to mobilise community responses to the COVID emergency. Formal methods of mobilisation tended to build upon existing associations such as religious institutions, resilience infrastructure and mother-toddler groups, but informal responses were more haphazard and spontaneous. However, there was an eruption of community spirit around horizontal ties. In our view this was more than a form of governmentality at distance, but neither was it opposition or protest. It opened up a “space” for different lines of communication and some relief from the top-down governmentality.

The forced turn towards localism during the first lockdown with radical lifestyle changes offered a glimpse into an alternative future that more neighbourliness, a slower pace of life, mindful appreciation of the surroundings and a low carbon footprint could offer. Even if this was rapidly forgotten later, it allowed spaces to emerge in an otherwise the pervasive biopolitical governmentality.

At a personal level, by contrast, personal communities of family and friends became de-spatialised as social relationships moved online, which also narrowed and intensified them, making it possible to connect with more distant people, but making it more difficult to make contact with casual or workplace acquaintances. In personal communities it was friendly rather than governmentality surviellance that dominated.

Things started to change as the lockdowns continued on and off for two years. Research suggests that the “rally round the flag” compliance of the first lockdown was eroded in later lockdowns as trust in government ebbed away. Opposition started to emerge (Schröder et al., 2022). By the summer of 2020, there was some evidence of either individual or collective disobedience as people started to increasingly disregard many of the regulations such as by flocking to popular sites once restrictions began to loosen. In Germany and Austria, weekly demonstrations by “Querdenker” represented a loose coalition of radical right, radical left, anti-state and alternative activists, and the downright loopy, some of them wearing silver foil helmets against the allegedly penetrating rays sent by Bill Gates and others. These protests gained ground by opposing the vaccination regimes imposed by the government. The frequent government policy changes tended to undermine confidence in regimes (Eurofound, 2021). Protests against lockdowns became more common in 2021 and were fuelled by the misinformation circulating on social media (Šrol et al., 2021; Eurofound, 2021) Indeed, it could be argued that the withering of peer interaction (Abrams et al., 2021) perhaps led to an increasing disconnect with reality as alternative thinking was fanned by retreat into rabbit holes of online (dis)information. At the same time critical voices could be discredited by being associated with these more extremist visions.

The data from elsewhere suggests that volunteering and community support might have been a temporary phenomenon produced by the lockdowns and that this kind of activity tailed off as lockdowns came and went. Meanwhile, the fall in well-being and trust in government on a number of different measures were measured across all European countries, so it is not clear that the invocation of national community spirit is sustainable (Eurofound, 2021). Indeed, it seems that the “rally round the flag” response materialised in the first lockdown, then fell way in subsequent ones.

We have raised the question of whether community represented an alternative to the increased governmentality of the coronavirus pandemic. The answer, based upon a small and specialised group of academics, suggests that whilst national community was strongly reinforced, and local communities did to some extent take over the surveillance of their members, the new kinds of local mobilisation also opened up spaces for horizontal lines of communication which went beyond simply an extension of governmentality. Compassionate communities emerged. However, these responses were very uneven across society, maybe reinforcing the advantage of already well organised localities. At the level of personal communities, the kind of surveillance taking place was supportive rather than repressive, although the limitations of digital communication may have limited the range of social connections and prevented the emergence of genuine critical discussion that comes with face-to-face communications. Isolation and the reliance on social media and online information sources may have facilitated the emergence of some forms of eccentric resistence.

This paper, based upon a small and untypical sample of high digital density academic households, but can help us to understand more about the personal consequences of how the pandemic was experienced.

### Ethics Approval

was granted by the University of Sussex on 30th March 2020. Each participant signed a consent form.
